# Supplementary material for: Drosophila COMPASS Complex Subunits Set1 and Ash2 Are Required for Oocyte Determination and Maintenance of the Synaptonemal Complex
Source: J Dev Biol. 2025 Aug 19;13(3):30. doi: 10.3390/jdb13030030 (PMC12372144; doi:10.3390/jdb13030030)
Supplement: Supplementary file 1 [file jdb-13-00030-s001.zip › Figure S1.pdf]

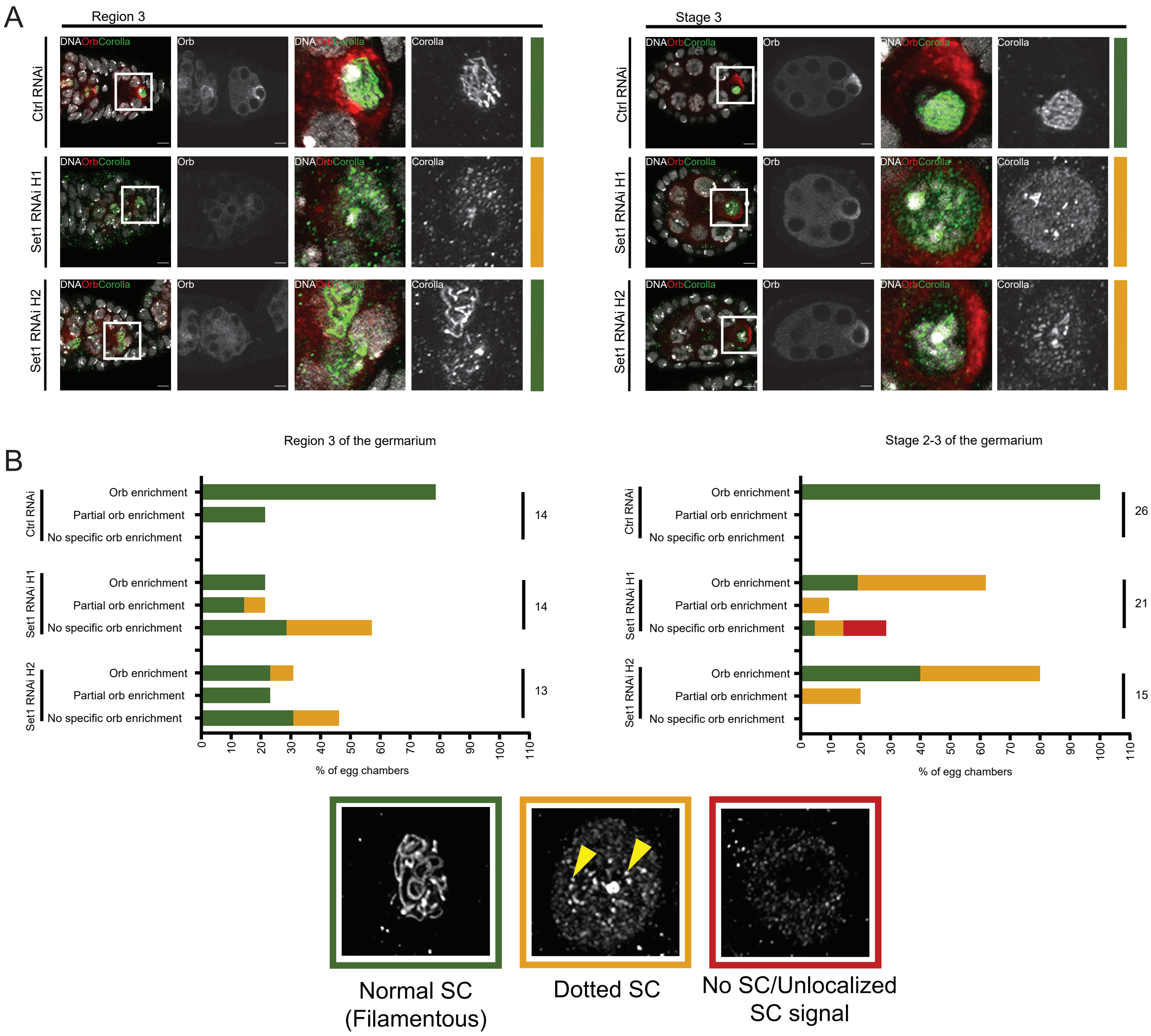
A
Region 3
Stage 3
Ctrl RNAi
Set1 RNAi H1
Set1 RNAi H2
DNA Orb Corolla
Orb
Corolla
B
Region 3 of the germarium
Stage 2-3 of the germarium
Orb enrichment
Partial orb enrichment
No specific orb enrichment
14
14
13
26
21
15
% of egg chambers
0
10
20
30
40
50
60
70
80
90
100
110
Normal SC (Filamentous)
Dotted SC
No SC/Unlocalized SC signal

Supplementary Figure S1. Two non-overlapping RNAi hairpins targeting Set1 demonstrate that Set1 is required for primary oocyte determination and maintenance of Synaptonemal Complex (SC) integrity.

(A) Immunofluorescence for Orb (oocyte marker; in red), Corolla (SC subunit; in green) and DNA (in grey) in control RNAi, Set1 RNAi (hairpin 1; H1) (dsRNA-HMS00581; BL33704) and Set1 RNAi (hairpin 2; H2) (dsRNA-HMS02179; BL40931) ovaries. Orb is shown in grey scale. Insets highlight SC assembly with Corolla shown in grey scale. Scale bar: 4,7μm. Images are partial z-projections of each egg chamber. **(B)** Correlation between SC assembly and Orb enrichment for control (mCherry RNAi), Set1 RNAi H1 and Set1 RNAi H2 in mid pachytene. For each egg chamber, Orb enrichment was assessed first, followed by evaluation of SC assembly. The number of scored egg chambers for each condition is indicated at the top of the corresponding bars.
