# Supplementary material for: Drosophila COMPASS Complex Subunits Set1 and Ash2 Are Required for Oocyte Determination and Maintenance of the Synaptonemal Complex
Source: J Dev Biol. 2025 Aug 19;13(3):30. doi: 10.3390/jdb13030030 (PMC12372144; doi:10.3390/jdb13030030)
Supplement: Supplementary file 1 [file jdb-13-00030-s001.zip › Tables S1 and S2.pdf]

Material and Methods

Table S1. *Drosophila* stocks used in this work

Drosophila stocks		
Gene	Bloomington stock number	TRiP RNAi construct
mCherry RNAi	35785	VALUIM20-mCherry.RNAi
Kdm5 RNAi	35706	GLV21071
Set1 RNAi H1	33704	HMS00581
Set1 RNAi H2	40931	HMS02179
ash2 RNAi	35388	GL00307

Table S2. Reagents and antibodies used in this work

Reagents				
Antibody	Source	Identifier	Working dilution	Reference
Orb	Developmental Studies Hybridoma Bank (Iowa City, United States of America)	Orb 4H8	1:30	PMID: 7523244
Orb	Developmental Studies Hybridoma Bank (Iowa City, United States of America)	Orb 6H4	1:30	PMID: 7523244
Corolla	Kind gift from Scott Hawley Lab		1:1000 or 1:2000	PMID: 24913682
H3K4me3	Active Motif (Carlsbad, California, United States of America)	39060	1:1000	
Alexa Fluor® 488 Donkey anti-rabbit IgG	BioLegend (San Diego, California, United States of America)	406416	1:200	
Alexa Fluor® 594 Goat anti-mouse IgG	BioLegend (San Diego, California, United States of America)	405326	1:200	

Resource	Source	Identifier
Paraformaldehyde	Electron Microscopy Science (Hatfield, Pennsylvania, United States of America)	Catalog no. 15714, lot no. 201005-50
NP-40	Sigma-Aldrich (St. Louis, Missouri, United States of America)	I8896-100ML, Lot no. MKBX5610V
Tween-20	Sigma-Aldrich (St. Louis, Missouri, United States of America)	P1379-25ML, Lot no. SLCH7513

Triton X-100	Alfa aesar by Thermo Fisher Scientific (Ward Hill, Massachusetts, United States of America)	A16046, lot U20G713
Bovine Serum Albumin	Sigma-Aldrich (St. Louis, Missouri, United States of America)	A7906-50G
Donkey serum	Sigma-Aldrich (St. Louis, Missouri, United States of America)	D9663-10ML
DAPI	Fluka (Buchs, Switzerland)	32670-5MG-F
VECTASHIELD	Vector Laboratories (Newark, California, United States of America)	H-100, lot no. ZH1108
